# High prevalence of *Pfdhfr*–*Pfdhps* quadruple mutations associated with sulfadoxine–pyrimethamine resistance in *Plasmodium falciparum* isolates from Bioko Island, Equatorial Guinea

**DOI:** 10.1186/s12936-019-2734-x

**Published:** 2019-03-26

**Authors:** Tingting Jiang, Jiangtao Chen, Hongxia Fu, Kai Wu, Yi Yao, Juan Urbano Monsuy Eyi, Rocio Apicante Matesa, Maximo Miko Ondo Obono, Weixing Du, Huabing Tan, Min Lin, Jian Li

**Affiliations:** 1 Department of Human Parasitology, School of Basic Medical Sciences, Department of Infectious Diseases, Renmin Hospital, Hubei University of Medicine, Shiyan, 442000 People’s Republic of China; 2 The Chinese Medical Aid Team To the Republic of Equatorial Guinea; Laboratory Medical Center, Huizhou Municipal Central Hospital, Huizhou, 516001 People’s Republic of China; 3 Clinical Laboratory, Taihe Hospital, Hubei University of Medicine, Shiyan, 442000 People’s Republic of China; 4 Department of Schistosomiasis and Endemic Diseases, Wuhan City Center for Disease Prevention and Control, Wuhan, People’s Republic of China; 5 Department of Medical Laboratory, Malabo Regional Hospital, Malabo, Equatorial Guinea; 6 Department of Histology and Embryology, Shantou University Medical College, Shantou, People’s Republic of China

**Keywords:** *Plasmodium falciparum*, Sulfadoxine–pyrimethamine, Anti-malarial drug resistance, Dihydropteroate synthase, Dihydrofolate reductase, Bioko Island

## Abstract

**Background:**

Sulfadoxine–pyrimethamine (SP) is recommended for intermittent preventive treatment of malaria in Africa. However, increasing SP resistance (SPR) affects the therapeutic efficacy of the SP. As molecular markers, *Pfdhfr* (dihydrofolate reductase) and *Pfdhps* (dihydropteroate synthase) genes are widely used for SPR surveillance. This study aimed to assess the prevalence of *Pfdhfr* and *Pfdhps* genes mutations and haplotypes in *Plasmodium falciparum* isolates collected from Bioko Island, Equatorial Guinea (EG).

**Methods:**

In total, 180 samples were collected in 2013–2014. The single nucleotide polymorphisms (SNPs) of the *Pfdhfr* and *Pfdhps* genes were identified with nested PCR and Sanger sequencing. The genotypes and linkage disequilibrium (LD) tests were also analysed.

**Results:**

Sequences of *Pfdhfr* and *Pfdhps* genes were obtained from 92.78% (167/180) and 87.78% (158/180) of the samples, respectively. For *Pfdhfr*, 97.60% (163/167), 87.43% (146/167) and 97.01% (162/167) of the samples carried N51I, C59R and S108N mutant alleles, respectively. The prevalence of the *Pfdhps* S436A, A437G, K540E, A581G, and A613S mutations were observed in 20.25% (32/158), 90.51% (143/158), 5.06% (8/158), 0.63% (1/158), and 3.16% (5/158) of the samples, respectively. In total, 3 unique haplotypes at the *Pfdhfr* locus and 8 haplotypes at the *Pfdhps* locus were identified. A triple mutation (CIRNI) in *Pfdhfr* was the most prevalent haplotype (86.83%), and a single mutant haplotype (SGKAA; 62.66%) was predominant in *Pfdhps*. A total of 130 isolates with 12 unique haplotypes were found in the *Pfdhfr* and *Pfdhps* combined haplotypes, 65.38% (85/130) of them carried quadruple allele combinations (CIRNI-SGKAA), whereas only one isolate (0.77%, 1/130) was found to carry the wild-type (CNCSI-SAKAA). For LD analysis, the *Pfdhfr* N51I was significantly associated with the *Pfdhps* A437G (P < 0.05).

**Conclusion:**

Bioko Island possesses a high prevalence of the *Pfdhfr* triple mutation (CIRNI) and *Pfdhps* single mutation (SGKAA), which will undermine the pharmaceutical effect of SP for malaria treatment strategies. To avoid an increase in SPR, continuous molecular monitoring and additional control efforts are urgently needed in Bioko Island, Equatorial Guinea.

## Background

Malaria is a major global public health concern particularly in sub-Saharan Africa, with 219 million cases of malaria and approximately 435,000 deaths in 2017 [1]. Most of the severe clinical cases and deaths were caused by *Plasmodium falciparum*. Furthermore, pregnant women and children under 5 years old are the main victims of falciparum malaria. To alleviate the global malaria burden in a susceptible population, sulfadoxine–pyrimethamine (SP) is recommended by the World Health Organization (WHO) for use as intermittent preventive treatment in pregnant women (IPTp) and infants (IPTi) in malaria-endemic regions [2].

Equatorial Guinea (EG) is a hyperendemic area of year-round malaria transmission [3], and the population is more frequently exposed to episodes of malaria [4]. Recent studies demonstrated *P. falciparum* parasites are the predominant species in EG, leading to approximately 291,700 cases in 2016; 15% of the deaths from this species were in children under 5 years old [5]. The authorities have deployed a series of measures that include effective anti-malarial drugs, vector control and case management for malaria control [6]. In 2004, The Bioko Island Malaria Control Project (BIMCP) was initiated on Bioko Island [7]. That project succeeded in reducing the infection rate, anaemia and child mortality [6]. Subsequently, similar measures have been adopted and were applied on mainland EG by the Equatorial Guinea Malaria Control Initiative (EGMCI) in 2007 [8]. In EG, SP has been used as a second-line treatment in cases of uncomplicated falciparum malaria for several decades. Furthermore, it was administered as the partner drug with artesunate as a first-line drug because of chloroquine treatment failure and as a malaria prophylaxis since 2004 [9], which may have led to *P. falciparum* isolates undergoing sustainable selection pressure. Soon afterwards, SP was replaced by artemisinin-based combination therapy (ACT) in response to widespread drug resistance in 2009, but it still remains the only choice for IPTp [10]. Of even greater concern, SP resistance (SPR) had already evolved in most African countries before SP was implemented as the recommended treatment. To ensure the prophylactic efficacy of this approach and support the national anti-malarial policy, large-scale screening and surveillance of SP drug resistance is highly recommended [11].

Targeting the *P. falciparum* enzymes dihydropteroate synthase (DHPS) and dihydrofolate reductase (DHFR), SP acts as a synergistic inhibitor of folate in the parasite [12, 13]. In vitro and in vivo studies have demonstrated that SPR is mainly conferred by amino acid point mutations at codons N51I, C59R, S108N, and I164L of *Pfdhfr* and S436A, A437G, K540E, A581G, and A613S of *Pfdhps* [14]. These hotspot mutations are suggested to be gradually displayed with the increase of SPR [15]. Many clinical failures have been reported after SP treatment was found in Africa [16–18]. Thus, an urgent need exists to continue monitoring and assessing resistance in *P. falciparum* populations when determining whether to administer this drug for prevention.

On Bioko Island, IPTp was introduced in 2004 [7, 9], and the Ministry of Health has implemented the use of two doses of SP during pregnancy and antenatal care, starting from the second trimester and 1 month apart [19]. An assessment of the prevalence of mutations in *P. falciparum* genes related to SPR on Bioko Island is needed to provide complementary information for this preventive strategy. In the current study, an assessment of the prevalence of the *Pfdhfr* and *Pfdhps* gene mutations and haplotypes was conducted on *P. falciparum* isolates collected from Bioko Island, EG.

## Methods

### Study area and samples collection

The study was performed in 2013–2014 on Bioko Island, the Insular Region of EG, where malaria is endemic and has continuous transmission throughout the year. Venous blood (3 ml) was collected from *P. falciparum*-infected patients and confirmed by thick and thin smears stained with diluted Giemsa. Additionally, positive blood spots were air dried, individually reserved in coded plastic bags with silica desiccant beads, and kept at room temperature for further molecular assessment.

### Ethics statement

The Ethics Committees of Malabo Regional Hospital on Bioko Island gave scientific and ethical permission (EGCNGD-071). Consent was obtained from all persons or their legal guardians before sample collection.

### DNA extraction and PCR

Genomic DNA was extracted from dried filtered bloodspots (DBS) by following the Chelex-100 extraction procedure described in the previous report [20]. The *Pfdhfr* and *Pfdhps* genes were amplified by nested PCR, and the conditions for amplification were as previously described [21]. The mutations of the *Pfdhfr* and *Pfdhps* genes in the amplified nested PCR products were purified and detected subsequently by Sanger sequencing (Genewiz, Soochow, China). All sequences were analysed using DNAstar (DNASTAR Inc., Madison, WI, USA).

### Data analysis

All data were analysed with SPSS 18 (SPSS Inc., Chicago, IL, USA). The percentages of single nucleotide polymorphisms (SNPs) and haplotypes were calculated with a 95% CI as described previously [22]. Differences in allele prevalence were compared using Pearson Chi square test or Fisher’s exact test, when conditions were appropriate. To determine the association between the SNPs of the *Pfdhfr* and *Pfdhps* genes, linkage disequilibrium (LD) tests were performed for each possible pair-wise SNP implicated as a drug-resistant marker in the two genes by calculating the D’ and *r*^2^ values using Haploview 4.2 software [23]. P values, less than 0.05 indicated significance.

## Results

### General information

In total, 180 isolates were evaluated. Then, 167 and 158 samples were successfully amplified, sequenced and genotyped for the *Pfdhfr* and *Pfdhps* genes, respectively. Of these successfully sequenced isolates, 130 sequences without any mixed types in *Pfdhfr* and *Pfdhps* were analysed for combined genotypes.

### Prevalence of individual point mutations in *Pfdhfr* and *Pfdhps*

A high prevalence of *Pfdhfr* mutant alleles was detected in the analysed samples. The two major mutations, N51I (97.60%; 163/167) and S108N (97.01%; 162/167), showed similar prevalence, followed by C59R (87.43%; 146/167). The C59R mutant allele showed lower prevalence compared to N51I and S108N (χ^2^ = 6.141, P = 0.013; χ^2^ = 6.082, P = 0.014). No mutation was identified at positions 50 and 164. The key mutation of *Pfdhps* linked to sulfadoxine resistance at codon A437G was predominant at 90.51% (143/158), while the prevalence of the S436A mutation was found to be 20.25% (32/158), and the K540E, A581G and A613S mutations were less frequent, occurring at rates of 5.06% (8/158), 0.63% (1/158) and 3.16% (5/158), respectively. The A437G mutation occurred at a significantly different rate compared with S436A (χ^2^ = 153.837, P < 0.001), K540E (χ^2^ = 234.34, P < 0.001), A581G (χ^2^ = 259.538, P < 0.001), and A613S (χ^2^ = 249.161, P < 0.001). Similar to A437G, the S436A occurred at a significantly different rate compared with K540E (χ^2^ = 17.929, P < 0.001), A581G (χ^2^ = 34.142, P < 0.001) and A613S (χ^2^ = 24.726, P < 0.001) (Table 1).

**Table 1:** Prevalence of *Pfdhfr* and *Pfdhps* SNPs in *Plasmodium falciparum* isolates from Bioko Island, Equatorial Guinea

Gene	SNP	Wild type	Mutation	Mixed type
		n (%, 95% CI)	n (%, 95% CI)	n (%, 95% CI)
Pfdhfr (n = 167)	51	3 (1.80, − 0.22 to 3.82)	163 (97.60, 95.28 to 99.92)	1 (0.60, − 0.57 to 1.77)
	59	12 (7.19, 3.27 to 11.11)	146 (87.43, 82.4 to 92.46)	9 (5.39, 1.97 to 8.81)
	108	3 (1.80, − 0.22 to 3.82)	162 (97.01, 94.43 to 99.59)	2 (1.20, − 0.45 to 2.85)
Pfdhps (n = 158)	436	115 (72.78, 65.84 to 79.72)	32 (20.25, 13.98 to 26.52)	11 (6.96, 2.99 to 10.93)
	437	12 (7.59, 3.46 to 11.72)	143 (90.51, 85.94 to 95.08)	3 (1.90, − 0.23 to 4.03)
	540	146 (92.41, 88.28 to 96.54)	8 (5.06, 1.64 to 8.48)	4 (2.53, 0.08 to 4.98)
	581	152 (96.20, 93.22 to 99.18)	1 (0.63, − 0.6 to 1.86)	5 (3.16, 0.43 to 5.89)
	613	153 (96.84, 94.11 to 99.57)	5 (3.16, 0.43 to 5.89)	0 (0.00)

*SNPs* single nucleotide polymorphisms, *n* number, *CI* confidence interval

### Prevalence of *Pfdhfr* and *Pfdhps* haplotypes

In the reconstitution of the haplotypes, 3 and 8 distinct genotypes were observed in *Pfdhfr* and *Pfdhps*, respectively, and mixed genotypes were also found in both genes. For *Pfdhfr*, only 1.2% (2/167) of the isolates were wild type CNCSI whereas 86.83% (145/167) carried the triple mutation CIRNI. The double mutant CICNI occurred with low prevalence at 5.99% (10/167). The overall prevalence of the mixed haplotypes was 5.99% (10/167) as follows: 0.6% (1/167) CNC/RSI, 4.19% (7/167) CIC/RNI, 0.6% (1/167) CIRS/NI, and 0.6% (1/167) CN/IC/RS/NI. For *Pfdhps*, the single mutated haplotype SGKAA was present in 62.66% (99/158) of the samples, followed by the double mutant haplotypes AGKAA in 10.76% (17/158), whereas only one isolate exhibited the triple mutated haplotype SGEGA. Of the remaining samples, 5.7% (9/158) harboured AAKAA, 4.43% (7/158) SGEAA, 0.63% (1/158) SGKAS, and 2.53% (4/158) AGKAS. The overall prevalence of mixed haplotypes was 11.39% (18/158) as follows: 0.63% (1/158) S/AAKAA, 3.8% (6/158) S/AGKAA, 1.9% (3/158) SGK/EAA, 0.63% (1/158) SGKA/GA, 1.27% (2/158) AGKA/GA, 0.63% (1/158) S/AGKA/GA, 1.9% (3/158) S/AA/GKAA, and 0.63% (1/158) SGK/EA/GA (Table 2).

**Table 2:** Prevalence of *Pfdhfr* and *Pfdhps* haplotypes in *Plasmodium falciparum* isolates from Bioko Island, Equatorial Guinea

Gene	Category	Haplotype	n (%, 95% CI)
Pfdhfr (n = 167)	Wild type	CNCSI	2 (1.20, − 0.45 to 2.85)
	Double mutant	CICNI	10 (5.99, 2.39 to 9.59)
	Triple mutant	CIRNI	145 (86.83, 81.7 to 91.96)
	Mixed type	CNC/RSI	1 (0.60, − 0.57 to 1.77)
		CIC/RNI	7 (4.19, 1.15 to 7.23)
		CIRS/NI	1 (0.60, − 0.57 to 1.77)
		CN/IC/RS/NI	1 (0.60, − 0.57 to 1.77)
Pfdhps (n = 158)	Wild type	SAKAA	2 (1.27, − 0.48 to 3.02)
	Single mutant	AAKAA	9 (5.70, 2.08 to 9.32)
		SGKAA	99 (62.66, 55.12 to 70.2)
	Double mutant	AGKAA	17 (10.76, 5.93 to 15.59)
		SGEAA	7 (4.43, 1.22 to 7.64)
		SGKAS	1 (0.63, − 0.6 to 1.86)
	Triple mutant	AGKAS	4 (2.53, 0.08 to 4.98)
		SGEGA	1 (0.63, − 0.6 to 1.86)
	Mixed type	S/AAKAA	1 (0.63, − 0.6 to 1.86)
		S/AGKAA	6 (3.80, 0.82 to 6.78)
		SGK/EAA	3 (1.90, − 0.23 to 4.03)
		SGKA/GA	1 (0.63, − 0.6 to 1.86)
		AGKA/GA	2 (1.27, − 0.48 to 3.02)
		S/AA/GKAA	3 (1.90, − 0.23 to 4.03)
		S/AGKA/GA	1 (0.63, − 0.6 to 1.86)
		SGK/EA/GA	1 (0.63, − 0.6 to 1.86)

*n* number, mutated alleles are underlined, *CI* confidence interval

### *Pfdhfr* and *Pfdhps* allele combinations

When the *Pfdhfr* and *Pfdhps* haplotypes were combined, 12 genotypes were verified and are shown in Table 3. Quadruple mutant haplotypes with a triple *Pfdhfr* and a single *Pfdhps* mutation (CIRNI-SGKAA) was the most common at 65.38% (85/130). One sample at the *Pfdhfr* and *Pfdhps* loci was fully a wild type. The second prevalent haplotype was CIRNI-AGKAA with a frequency of 12.31% (16/130). The quintuple mutation (CIRNI-SGEAA) and sextuple mutation (CIRNI-SGEGA) were found in 4.62% (6/130) and 0.77% (1/130) of the isolates, respectively. The occurrence of other combined haplotypes was generally low: 0.77% (1/130) CNCSI-SGKAA, 4.62% (6/130) CICNI-SGKAA, 0.77% (1/130) CIRNI-SAKAA, 0.77% (1/130) CICNI-SGEAA, 5.38% (7/130) CIRNI-AAKAA, 0.77% (1/130) CIRNI-SGKAS, and 3.08% (4/130) CIRNI-AGKAS.

**Table 3:** Prevalence of *Pfdhfr* and *Pfdhps* allele combinations in *Plasmodium falciparum* isolates from Bioko Island, Equatorial Guinea

Gene	Category	Haplotype	n (%, 95% CI)
Pfdhfr/Pfdhps (n = 130)	Wild type	CNCSI-SAKAA	1 (0.77, − 0.73 to 2.27)
	Single mutant	CNCSI-SGKAA	1 (0.77, − 0.73 to 2.27)
	Triple mutant	CICNI-SGKAA	6 (4.62, 1.01 to 8.23)
		CIRNI-SAKAA	1 (0.77, − 0.73 to 2.27)
	Quadruple mutant	CICNI-SGEAA	1 (0.77, − 0.73 to 2.27)
		CIRNI-AAKAA	7 (5.38, 1.5 to 9.26)
		CIRNI-SGKAA	85 (65.38, 57.2 to 73.56)
		CIRNI-AGKAA	16 (12.31, 6.66 to 17.96)
	Quintuple mutant	CIRNI-SGEAA	6 (4.62, 1.01 to 8.23)
		CIRNI-SGKAS	1 (0.77, − 0.73 to 2.27)
	Sextuple mutant	CIRNI-AGKAS	4 (3.08, 0.11 to 6.05)
		CIRNI-SGEGA	1 (0.77, − 0.73 to 2.27)

*n* number, *CI* confidence interval, mutated alleles are underlined

### Linkage disequilibrium (LD) test for *Pfdhfr* and *Pfdhps* haplotypes

**Fig. 1:**
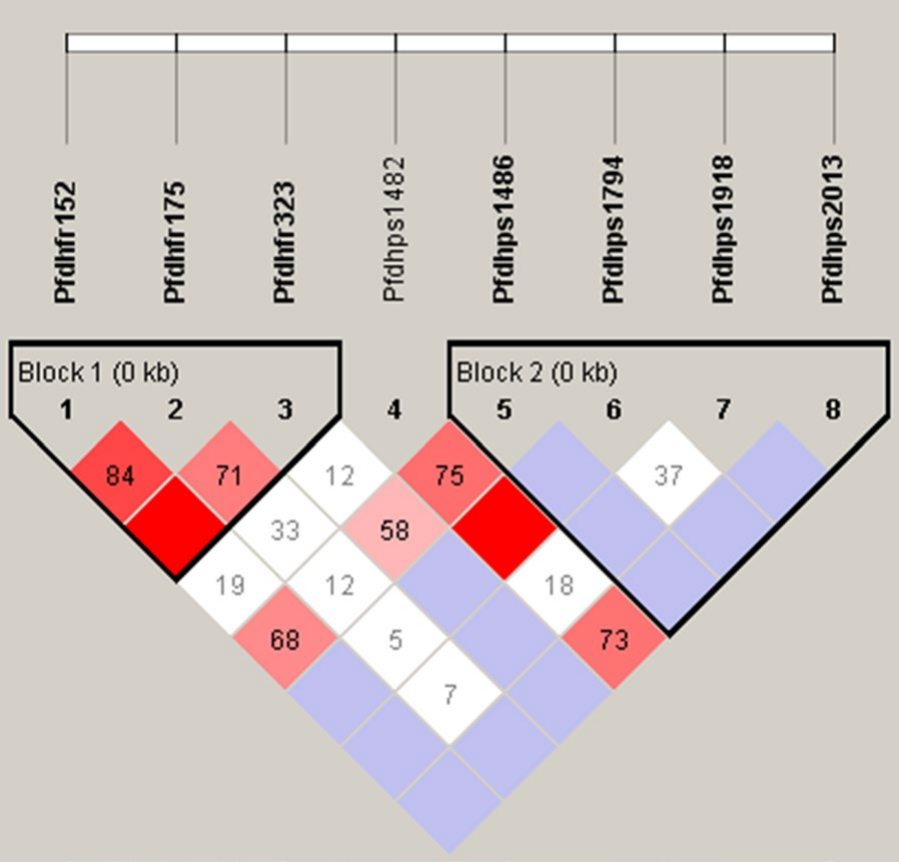
Linkage disequilibrium (LD) between pairs of single nucleotide polymorphisms (SNPs) located in *Pfdhfr* and *Pfdhps* and implicated in drug resistance for *Plasmodium falciparum* isolates from Bioko Island, Equatorial Guinea. For the *Pfdhfr* gene, base substitution mutations of T152A, T175C, and G323A are related to single amino acid mutations of N51I, C59R, and S108N, respectively. Similarly, the T1482G, C1486G, A1794G, and G2013T in the *Pfdhps* gene are related to mutations of S436A, A437G, K540E, and A613S, respectively. According to the four-gamete test, these SNPs are divided into two blocks (black frame). The number in the square indicates a D’ value. The square with dark red and light red indicates a linkage that was statistically significant (P < 0.05). The square with Cambridge blue indicates a linkage is present but is not statistically significant (P > 0.05). The square with white indicates no linkage is present

The LD pattern for each SNP in the *Pfdhfr* and *Pfdhps* genes was assessed (Fig. 1). For the *Pfdhfr* gene, base substitution mutations of T152A, T175C and G323A were related to the single amino acid mutations of N51I, C59R, S108N, respectively. Similarly, the T1482G, C1486G, A1794G, and G2013T in the *Pfdhps* gene indicated mutations of S436A, A437G, K540E, and A613S, respectively.

Several statistically significant associations were found among the SNPs located in both the *Pfdhfr* and *Pfdhps* genes (Fig. 1). For the *Pfdhfr* gene, T152A, T175C and G323 were in an LD block. The T152A (N51I) were significantly associated with the SNPs (T175C, C59R and G323A, S108N) with a D’ value of 0.84 (P < 0.05) and 1.0 (P < 0.05), respectively. Similarly, the T175C was significantly associated with the G323A (0.71, P < 0.05). For the *Pfdhps* gene, T1482G, C1486G, A1794G, and G2013T formed an LD block. The sole SNP (T1482G, S436A) were significantly associated with the SNPs (C1486G, A437G; A1794G, K540E; and G2013T, A613S) with D’ values of 0.75, 1.0 and 0.73, respectively. The SNP (T152A) of the *Pfdhfr* gene coding N51I was significantly associated with the C1486G, and the value is 0.68. No such association was detected in the other SNPs of either the *Pfdhfr* or *Pfdhps* genes.

## Discussion

The rapid and widespread development of anti-malarial drug resistance is directly influencing and hindering the process of malaria control, prevention and elimination [24]. Surveillance with molecular markers has allowed the early detection of drug resistance susceptibility and may provide fundamental information for drug policy [25]. The current study displays the mutations and haplotypes of the *Pfdhfr* and *Pfdhps* genes from isolates collected from the general population on Bioko Island, thus allowing the degree of SPR in this malaria hotspot to be inferred.

The results demonstrate that *Pfdhfr* polymorphism associated with SPR persists at high frequency. A high prevalence of the *Pfdhfr* N51I mutation in 97.60% and the S108N mutation in 97.01% of the samples was found among the *P. falciparum* population on Bioko Island (Table 1), and these mutations also had been found at a very high level (97.9 and 99.1%, respectively) in the Democratic Republic of Congo (DRC) in 2008 [26]. For C59R, the level was significantly lower than for N51I and S108N, similar to observations in the mainland of EG [4]. Like neighbouring countries, *Pfdhfr* I164L, which is related to high-grade SPR, has been reported at low proportions (1.4%) in rural areas of the EG mainland [4, 27]. Fortunately, this mutation was not found in any isolates within the study. Although the mutations of *Pfdhfr* C50R and I164L are not found in the present data, the high prevalence of three well-characterized mutations in *Pfdhfr* (N51I, C59R, S108N) indicate the *P. falciparum* isolates from Bioko island display high pyrimethamine resistance that needs to be addressed by the EGMCI. For the *Pfdhfr* haplotypes, 86.83% of the isolates carried the *Pfdhfr* triple mutation (CIRNI) (Table 2) and was reported in 80% of *P. falciparum* infections in 2005 from the mainland of EG, 100% in 2005 in Cameroon [28], and 72.4% in Gabon [29]. This triple mutation is an important SPR indicator, but its detrimental effects may be largely compromised by an absence of the *Pfdhfr* I164L mutation [30, 31]. The frequency of the *Pfdhfr* double mutant CICNI was 5.99% (Table 2), and this genotype has a lesser degree of resistance compared with the triple mutation CIRNI [29]. For the dominant mutant haplotype CIRNI (86.83%) and the double mutant haplotype CICNI (5.99%), the results are consistent with previous studies in EG and Central Africa [4, 10, 32, 33]. If the CIRNI haplotype is found concurrently with the *Pfdhps* mutations, it is associated with a high level of resistance [34]. The reported prevalence of the *Pfdhfr* triple mutation was also lower than those previously reported at the site where the proportion of the *Pfdhfr* triple mutation reached a frequency of 97%. Only 1.2% of the isolates (2/167) were a pure *Pfdhfr* wild type (CNCSI) (Table 2). The results indicate that almost all samples collected harbour pyrimethamine resistance.

Compared with the mutations of the *Pfdhfr* gene, the mutations of the *Pfdhps* gene exhibit a relatively low prevalence, except for the A437G mutation (90.51%, 143/158) (Table 1), which is also common in other EG regions and several African countries [27, 31, 35]. This mutation has been reported to occupy the key position of the initial mutation of sulfadoxine resistance, and its resistance increases along with the augmentation of other mutations in *Pfdhps* [36]. Although the prevalence of S436A is significantly lower than that of A437G, it is higher than for other mutations, including K540E, A581G and A613S. In Central Africa, the *Pfdhps* K540E mutation was less prevalent, which was also confirmed in this study (5.06%, 8/158) (Table 1). This mutation is more common in East Africa, particularly in Tanzania [37] and Uganda [17]. The WHO has recommended that IPT with SP should be abandoned in areas where the K540E mutation has been detected at > 95% and *Pfdhps* the A581G mutations are detected at > 10% because it could be ineffective [11]. Fortunately, only 5.06% (5/158) of the isolates showed the *Pfdhps* K540E mutation, and 0.63% (1/158) of the isolates harboured the A581G mutation in current survey (Table 1). The relatively low prevalence of these mutations suggests that IPT-SP can possibly be efficacious on Bioko Island, EG. The A613S mutations were detected in 3.16% (5/158) of the isolates, which is consistent with reports in Central African countries, including the DRC [27] and Cameroon [29]. For the *Pfdhps* haplotype, the single-mutant SGKAA haplotype predominates in our results (62.66%) (Table 2), similar to observations made in Gabon [38] and the DRC [39]. AGKAA is present in 10.76% of the isolates (Table 2), and an increased trend was detected in Gabon between 2013 and 2014 [38]. Parasites with double- and triple-mutant *Pfdhps* haplotypes were observed at a low frequency (Table 2), suggesting a low tendency in the emergence and development of the sulfadoxine resistance alleles.

The combination of the *Pfdhfr* and *Pfdhps* mutant alleles generated 12 different haplotypes in the present survey (Table 3). Only one wild-type haplotype (CNCSI-SAKAA) was found in this study (Table 3). The quadruple mutant (CIRNI-SGKAA) was predominant, with a prevalence of 65.38% (Table 3), which is higher than reports from mainland EG (54%) [4]. The saturation of the *Pfdhfr* triple mutants could further induce the *Pfdhps* mutants, and thus, the presence of quadruple mutants (CIRNI-SGKAA) was common [40]. Although quintuple mutant genotypes (CIRNI-SGEAA) are highly linked to SP failure [34], this mutant was detected at a rate of 4.62% (Table 3). WHO recommends surveillancefor this genotype and inhibition of IPTp-SP when the prevalence of this quintuple mutant exceeds 50% [31]. To date, this quintuple mutant is less than 10% in other areas of EG [4, 10]. Previous in vitro studies demonstrated that the quadruple mutant (CIRNI-SGKAA) has a less deleterious effect on SP-IPT than the quintuple mutant genotypes (CIRNI-SGEAA) [41]. Notably, the ‘super resistant’ alleles (CIRNI-SGEGA) may render SP ineffective [42], but these were detected in only one isolate. Although this occurrence is low, sustainable monitoring for SPR and avoiding the growth of super resistance alleles are still critical.

Although the LD analysis of the SNPs between the *Pfdhfr* and *Pfdhps* genes showed a strong linkage between N51I and A437G, those main SNPs of the *Pfdhfr* and *Pfdhps* genes form two independent LD blocks, respectively. These results indicate that the mutations located in the *Pfdhfr* and *Pfdhps* genes have relative independence. However, combined chemotherapy will likely lead to the occurrence and progress of resistance gene mutations even though the *Pfdhfr* and *Pfdhps* genes are located on different chromosomes [40]. For the *Pfdhfr* gene, T152A, T175C, and G323A develop as a block. When distributed in the *Pfdhfr* gene, these SNPs exhibit strong linkage, particularly of N51I and S108N (D’: 0.71–1, P < 0.05). For the *Pfdhps* gene, the T1482G, C1486G, A1794G, and G2013T were found in an LD block. Although the SNPs in *Pfdhps* gene show weak linkage and no significant differences (P > 0.05), strong linkages were also commonly detected from S436A and other mutations, including A437G, K540E and A613S. Notably, the study had weaknesses, including the small sample size and the lack of full-length DNA sequences for the *Pfdhfr* and *Pfdhps* genes. In the present study, a 594-bp fragment of the *Pfdhfr* and a 711-bp fragment of the *Pfdhps* gene were amplified, based on previous study [21]. The sequences from these two fragments provide only limited information for LD analysis. Thus, the complete nucleotide sequences from the *Pfdhfr* and *Pfdhps* genes and the microsatellite loci flanking these genes [43] need to be amplified and genotyped in further study. Genetic diversity information and differentiation data from microsatellite loci flanking the *Pfdhfr* and *Pfdhps* genes will demonstrate whether the *P. falciparum* isolates have ever undergone selection in response to SP and may provide valuable information to solve anti-malarial drug resistance, particularly SPR.

## Conclusions

The results of this study indicate that this area had a high prevalence of the *Pfdhfr* triple mutation (CIRNI) and the *Pfdhps* single mutation (SGKAA), which could undermine the efficacy of SP for chemoprevention strategy. To avoid increases in SPR, continuous molecular monitoring and additional control efforts are urgently needed.

## References

[1] WHO (2018). World Malaria Report 2018.

[2] Gosling RD, Cairns ME, Chico RM, Chandramohan D (2010). Intermittent preventive treatment against malaria: an update. Expert Rev Anti Infect Ther..

[3] Benito A, Roche J, Molina R, Amela C, Alvar J (1994). Application and evaluation of QBC malaria diagnosis in a holoendemic area. Appl Parasitol.

[4] Berzosa P, Estebancantos A, García L, González V, Navarro M, Fernández T (2017). Profile of molecular mutations in pfdhfr, pfdhps, pfmdr1, and pfcrt genes of *Plasmodium falciparum* related to resistance to different anti-malarial drugs in the Bata District (Equatorial Guinea). Malar J..

[5] Guerra M, de Sousa B, Ndong-Mabale N, Berzosa P, Arez AP (2018). Malaria determining risk factors at the household level in two rural villages of mainland Equatorial Guinea. Malar J..

[6] Kleinschmidt I, Sharp B, Benavente LE, Schwabe C, Torrez M, Kuklinski J (2006). Reduction in infection with *Plasmodium falciparum* one year after the introduction of malaria control interventions on Bioko Island, Equatorial Guinea. Am J Trop Med Hyg.

[7] Kleinschmidt I, Torrez M, Schwabe C, Benavente L, Seocharan I, Jituboh D (2007). Factors influencing the effectiveness of malaria control in Bioko Island, equatorial Guinea. Am J Trop Med Hyg.

[8] Ridl FC, Bass C, Torrez M, Govender D, Ramdeen V, Yellot L (2008). A pre-intervention study of malaria vector abundance in Rio Muni, Equatorial Guinea: their role in malaria transmission and the incidence of insecticide resistance alleles. Malar J..

[9] Charle P, Berzosa P, Descalzo MA, Lucio AD, Raso J, Obono J (2014). Efficacy of artesunate + sulphadoxine–pyrimethamine (AS + SP) and amodiaquine + sulphadoxine–pyrimethamine (AQ + SP) for uncomplicated falciparum malaria in Equatorial Guinea (Central Africa). J Trop Med..

[10] Guerra M, Neres R, Salgueiro P, Mendes C, Ndongmabale N, Berzosa P (2016). *Plasmodium falciparum* genetic diversity in continental Equatorial Guinea before and after Introduction of artemisinin-based combination therapy. Antimicrob Agents Chemother.

[11] WHO (2013). Policy brief for the implementation of intermittent preventive treatment of malaria in pregnancy using sulfadoxine-pyrimethamine (IPTp-SP).

[12] Triglia T, Wang P, Sims PF, Hyde JE, Cowman AF (1998). Allelic exchange at the endogenous genomic locus in *Plasmodium falciparum* proves the role of dihydropteroate synthase in sulfadoxine-resistant malaria. EMBO J.

[13] Zolg JW, Plitt JR, Chen GX, Palmer S (1989). Point mutations in the dihydrofolate reductase-thymidylate synthase gene as the molecular basis for pyrimethamine resistance in *Plasmodium falciparum*. Mol Biochem Parasitol.

[14] Felix KK, Damien B, Anna F, Michael N, Christevy V, Francine N (2015). High prevalence of sulphadoxine–pyrimethamine resistance-associated mutations in *Plasmodium falciparum* field isolates from pregnant women in Brazzaville, Republic of Congo. Infect Genet Evol..

[15] Mita T, Ohashi J, Venkatesan M, Marma ASP, Nakamura M, Plowe CV (2014). Ordered accumulation of mutations conferring resistance to sulfadoxine-pyrimethamine in the *Plasmodium falciparum* parasite. J Infect Dis.

[16] Uchenna Anthony U, Obi SN, Onah HE, Ugwu EOV, Leonard Ogbonna A, Chioma Roseline U (2012). The impact of intermittent preventive treatment with sulfadoxine-pyrimethamine on the prevalence of malaria parasitaemia in pregnancy. Trop Doct.

[17] Braun V, Rempis E, Schnack A, Decker S, Rubaihayo J, Tumwesigye NM (2015). Lack of effect of intermittent preventive treatment for malaria in pregnancy and intense drug resistance in western Uganda. Malar J..

[18] Desai M, Gutman J, Taylor SM, Wiegand RE, Khairallah C, Kayentao K (2015). Impact of sulfadoxine-pyrimethamine resistance on effectiveness of intermittent preventive therapy for malaria in pregnancy at clearing infections and preventing low birth weight. Clin Infect Dis.

[19] Rehman AM, Mann AG, Schwabe C, Reddy MR, Roncon Gomes I, Slotman MA (2013). Five years of malaria control in the continental region, Equatorial Guinea. Malar J..

[20] Li J, Chen J, Xie D, Montenguba S, Eyi JUM, Matesa RA (2014). High prevalence of pfmdr1 N86Y and Y184F mutations in *Plasmodium falciparum* isolates from Bioko island, Equatorial Guinea. Pathog Glob Health..

[21] Pearce RJ, Chris D, Daniel C, Frank M, Cally R (2003). Molecular determination of point mutation haplotypes in the dihydrofolate reductase and dihydropteroate synthase of *Plasmodium falciparum* in three districts of northern Tanzania. Antimicrob Agents Chemother.

[22] Li J, Chen J, Xie D, Eyi UM, Matesa RA, Obono MMO (2015). Molecular mutation profile of Pfcrt and Pfmdr1 in *Plasmodium falciparum* isolates from Bioko Island, Equatorial Guinea. Infect Genet Evol..

[23] Patel P, Bharti PK, Bansal D, Ali NA, Raman RK, Mohapatra PK (2017). Prevalence of mutations linked to antimalarial resistance in *Plasmodium falciparum* from Chhattisgarh, Central India: a malaria elimination point of view. Sci Rep..

[24] Xu C, Wei Q, Yin K, Sun H, Li J, Xiao T (2018). Surveillance of Antimalarial Resistance Pfcrt, Pfmdr1, and Pfkelch13 Polymorphisms in African *Plasmodium falciparum* imported to Shandong Province, China. Sci Rep..

[25] Vestergaard LS, Ringwald P (2007). Responding to the challenge of antimalarial drug resistance by routine monitoring to update national malaria treatment policies. Am J Trop Med Hyg.

[26] Mobula L, Lilley B, Tshefu AK, Rosenthal PJ (2009). Resistance-mediating polymorphisms in *Plasmodium falciparum* infections in Kinshasa, Democratic Republic of the Congo. Am J Trop Med Hyg.

[27] Nkoli Mandoko P, Rouvier F, Matendo Kakina L, Moke Mbongi D, Latour C, Losimba Likwela J (2018). Prevalence of *Plasmodium falciparum* parasites resistant to sulfadoxine/pyrimethamine in the Democratic Republic of the Congo: emergence of highly resistant pfdhfr/pfdhps alleles. J Antimicrob Chemother.

[28] Menemedengue V, Sahnouni K, Basco L, Tahar R (2011). Molecular epidemiology of malaria in Cameroon. XXX. sequence analysis of *Plasmodium falciparum* ATPase 6, dihydrofolate reductase, and dihydropteroate synthase resistance markers in clinical isolates from children treated with an artesunate-sulfadoxine-pyrimethamine combination. Am J Trop Med Hyg..

[29] Ngomo JMN, Mawilimboumba DP, M’Bondoukwe NP, Ella RNN, Akotet MKB (2016). Increased prevalence of mutant allele Pfdhps 437G and Pfdhfr triple mutation in *Plasmodium falciparum* isolates from a rural area of Gabon, three years after the change of malaria treatment policy. Malar Res Treat..

[30] Rain AN, Azrina NN, Jenarun J, Hakim SL, Hasidah MS, Zakiah I (2013). High prevalence of mutation in the *Plasmodium falciparum* dhfr and dhps genes in field isolates from Sabah, Northern Borneo. Malar J..

[31] Grais RF, Laminou IM, Woimesse L, Makarimi R, Bouriema SH, Langendorf C (2018). Molecular markers of resistance to amodiaquine plus sulfadoxine–pyrimethamine in an area with seasonal malaria chemoprevention in south central Niger. Malar J..

[32] Mendes C, Salgueiro P, Gonzalez V, Berzosa P, Benito A, do Rosario VE (2013). Genetic diversity and signatures of selection of drug resistance in Plasmodium populations from both human and mosquito hosts in continental Equatorial Guinea. Malar J..

[33] Chauvin P, Menard S, Iriart X, Nsango SE, Tchioffo MT, Abate L (2015). Prevalence of *Plasmodium falciparum* parasites resistant to sulfadoxine/pyrimethamine in pregnant women in Yaounde, Cameroon: emergence of highly resistant pfdhfr/pfdhps alleles. J Antimicrob Chemother.

[34] Salem MS, Lekweiry KM, Bouchiba H, Pascual A, Pradines B, Boukhary AO (2017). Characterization of *Plasmodium falciparum* genes associated with drug resistance in Hodh Elgharbi, a malaria hotspot near Malian-Mauritanian border. Malar J..

[35] Osarfo J, Tagbor H, Magnussen P, Alifrangis M (2018). Molecular markers of *Plasmodium falciparum* drug resistance in parasitemic pregnant women in the middle forest belt of Ghana. Am J Trop Med Hyg.

[36] Wernsdorfer WH, Noedl H (2003). Molecular markers for drug resistance in malaria: use in treatment, diagnosis and epidemiology. Curr Opin Infect Dis..

[37] Kavishe RA, Kaaya RD, Nag S, Krogsgaard C, Notland JG, Kavishe AA (2016). Molecular monitoring of *Plasmodium falciparum* super-resistance to sulfadoxine–pyrimethamine in Tanzania. Malar J..

[38] Voumbo-Matoumona DF, Kouna LC, Madamet M, Maghendji-Nzondo S, Pradines B, Lekana-Douki JB (2018). Prevalence of *Plasmodium falciparum* antimalarial drug resistance genes in Southeastern Gabon from 2011 to 2014. Infect Drug Resist..

[39] Baraka V, Delgadoratto C, Nag S, Ishengoma DS, Madebe RA, Mavoko HM (2017). Different origin and dispersal of sulphadoxine-resistant *Plasmodium falciparum* haplotypes between Eastern Africa and Democratic Republic of Congo. Int J Antimicrob Agents.

[40] Zhang Y, Yan H, Wei G, Han S, Huang Y, Zhang Q (2014). Distinctive origin and spread route of pyrimethamine-resistant *Plasmodium falciparum* in Southern China. Antimicrobial Agents Chemother..

[41] Kaingonadaniel EPS, Gomes LR, Gama BE, Almeidadeoliveira NK, Fortes F, Ménard D (2016). Low-grade sulfadoxine–pyrimethamine resistance in *Plasmodium falciparum* parasites from Lubango, Angola. Malar J..

[42] Hemming-Schroeder E, Umukoro E, Lo E, Fung B, Tomás-Domingo P, Zhou G (2018). Impacts of antimalarial drugs on *Plasmodium falciparum* drug resistance markers, western Kenya, 2003–2015. Am J Trop Med Hyg.

[43] Alam MT, de Souza DK, Vinayak S, Griffing SM, Poe AC, Duah NO (2011). Selective sweeps and genetic lineages of *Plasmodium falciparum* drug -resistant alleles in Ghana. J Infect Dis.

